# Sports and Functional Training Improve a Subset of Obesity-Related Health Parameters in Adolescents: A Randomized Controlled Trial

**DOI:** 10.3389/fpsyg.2020.589554

**Published:** 2021-01-21

**Authors:** Braulio Henrique Magnani Branco, Isabela Ramos Mariano, Leonardo Pestillo de Oliveira, Sônia Maria Marques Gomes Bertolini, Fabiano Mendes de Oliveira, Cynthia Gobbi Alves Araújo, Kristi Adamo

**Affiliations:** ^1^Research Group in Physical Education, Physiotherapy, Sports, Nutrition and Performance, Unicesumar University, Maringa, Brazil; ^2^Graduate Program in Health Promotion, Unicesumar University, Maringa, Brazil; ^3^Faculty of Health Sciences, School of Human Kinetics, University of Ottawa, Ottawa, ON, Canada; ^4^Graduate Program in Biological Sciences, State University of Maringa, Maringa, Brazil

**Keywords:** adolescent health, cardiometabolic risk in adolescents, exercise physiology, multi-professional research, psychophysiological habituation

## Abstract

To investigate the effects of two different modes of physical activity on body composition, physical fitness, cardiometabolic risk, and psychological responses in female adolescents participating in a multi-disciplinary program. The 12-week randomized intervention included 25-adolescents with overweight divided into two groups: sports practice-SPG and functional training-FTG. The SPG intervention was divided into three sports: basketball, handball, and futsal. SPG participants performed one sport 3-times/week, over the course of 1 month. The FTG performed concurrent exercises 3-times/week. This study was registered in Clinical Trials Registry Platform under number: RBR-45ywtg and registered in Local Ethics Committee number: 2,505.200/2018. The intensity of physical exercises-PE was matched between groups by the rating of perceived exertion. The primary outcome was body composition, and secondary outcomes were physical fitness, cardiometabolic risk, and psychological responses. There was a significant time-effect for body mass, body mass index, and low-density lipoprotein (LDL-c), all being reduced. There were increases over time for musculoskeletal mass, aerobic fitness, and high-density lipoprotein (HDL-c) (*p* < 0.05). There was a group time interaction with body fat percentage being lower post-intervention in the SPG (*p* < 0.05). No significant differences were observed for the other variables. Both physical activity models were effective in improving a subset of obesity-related health parameters. The findings should be extended by further investigation using more sophisticated measures of energy expenditure.

**Clinical Trial Registration:** https://ensaiosclinicos.gov.br/, identifier: RBR-45ywtg.

## Introduction

In the 21st century, obesity has reached pandemic proportions across all age groups (Nyberg et al., 2018). While fundamentally an issue of energy balance, the contributions of factors related to nutrition, psychology, physiology and metabolism, physical, activity, socioeconomic status, among others, make obesity treatment, and prevention highly complex (Todd et al., 2015). Modern behaviors' which heavily contribute to weight gain are the reduction of physical activity levels (Morano et al., 2016; Kebbe et al., 2017; Elmesmari et al., 2018), and an increase of processed and ultra-processed foods in the diets of adolescents (Louzada et al., 2015). In view of the multi-factorial nature of obesity, the development of multi-disciplinary interventions to combat obesity is necessary for all ages, including adolescents (Lopera et al., 2016; Morano et al., 2016; Bianchini et al., 2017; Freitas et al., 2017; Magnani Branco et al., 2018; Branco et al., 2019). The multi-professional interventions could be conducted with theoretical and practical interventions to promote health literacy and improve physical activity (PA). Furthermore, these intervention programs provide accessible resources to support public and private systems in managing obesity and minimizing expenses from hospital treatment of this disease and its associated comorbidities (Magnani Branco et al., 2018; Branco et al., 2019).

Recent evidence published by Branco et al. (2018, 2019) compared different PA modalities throughout the multi-professional approach model that includes PA, nutritional, and psychological counseling. This group concluded that models of PA, when equalized for rating of perceived exertion (RPE), muscle groups utilized, and effort-pause ratio (i.e., when the order of exercises are changed), time session, and frequency, tend to show similar responses in anthropometrical parameters, physical fitness, and lipid profile among others. The HEARTY Study, undertaken by Sigal et al. (2014) found that a combined exercise program that included resistance plus aerobic training resulted in greater reductions in anthropometric parameters in adolescents with obesity when compared with an aerobic only, or resistance only exercise intervention. Despite the HEARTY study's results, it is challenging to establish what PA program is more efficacious because the interventions used were not matched for volume and intensity (Andreato et al., 2019). Thus, to define what approach is more suited to promote weight-loss it is imperative to standardize intensity, volume, and frequency of the PA sessions.

Similar to HEARTY, the study by Lopera et al. (2016) administered a multi-disciplinary intervention focusing on the treatment of obesity in adolescents by comparing water- vs. land-based exercise training. Participants in both intervention groups responded similarly; while the number of exercise sessions and length of sessions were consistent between groups, the RPE (proxy for intensity) was not equalized. Indeed, different exercise intervention modalities were compared by Lopera et al. and participants responded comparably, but a relevant aspect to be considered is the equalization of intensity between interventions. A simple, non-invasive method to control the intensity of sessions is by using RPE, a factor not measured in the above-mentioned study.

To compare the responses between the groups, or to verify the efficacy of different PA modalities, the use of some method of controlling the sessions (such as RPE–perceived effort, time per session and exercise frequency per week) are needed. Previous acute studies (Zeni et al., 1996; Moyna et al., 2001) identified different energy expenditure in exercises conducted with the same RPE. Over time, this process could show differences in the weight-loss process. In the long term, participating in an exercise modality perceived to be less challenging, but that burns equivalent calories to one that feels more exertive may be an excellent strategy for keeping adolescents engaged. Thus, testing the impact of different exercise interventions, equalized for RPE, on the parameters related to weight-loss, fitness, and cardiometabolic health, as well as adherence and wellness in the adolescents, is important.

It is currently unknown if the health outcome responses are comparable between different models of RPE-matched PA as part of a multi-professional obesity treatment program. Consequently, the main objective of this study is to investigate the effects of two different models of PA on body composition, health-related physical fitness, psychological parameters, and cardiometabolic risk in adolescents participating in a multi-disciplinary obesity treatment program. It is hypothesized that the responses observed will be similar, as the two intervention models are equalized for perceived intensity (RPE), and large muscle groups are being used during both sets of intervention activities.

## Methods

### Multi-Disciplinary Intervention Systematization

For this 2-arm, parallel randomized controlled trial, 43 female adolescents were recruited from 2 schools and 2 Basic Health Units near the University, through posters, and meetings with parents or guardians. The inclusion criteria were as follows: (1) between 12 and 17 years of age; (2) classified as overweight or obesity based on Cole and Lobstein criteria (Cole and Lobstein, 2012); (3) capable of a participating in the different interventions for 12 weeks (3 times per week, ~2.5 h per session). The exclusion criteria were (1) engagement in structured PA outside of the intervention; (2) cognitive impairment that reduces the capacity to execute physical exercises or understand the theoretical classes; (3) physical disability or illness making participation inadvisable; (4) currently engaged in dietary restriction; (5) use of psychotropic or appetite-regulating medicine; (6) lack of adherence to intervention. Consented participants were randomized into two groups using a random numbers generator (www.random.org/): Sports Practice Group (SPG) and Functional Training Group (FTG). The SPG engaged in three different sports (basketball, handball, and futsal) over the course of the 12-week intervention. Participants focused on one sport, 3 times a week over 1 month, and then moved to the next sport. The FTG performed a structured exercise program comprised of two training programs, i.e., A and B, alternating each training session performed 3 times a week. More information about training programs can be found in **Table 2**.

Both groups of adolescents received theoretical and practical lessons from a team of physical education teachers, nutritionists, and psychologists. All activities started with theoretical classes and were performed in follow-up practical classes. The description of the different intervention groups is explained in detail below. The PA sessions were conducted 3 times a week; theoretical nutrition classes of 2 times per week, and psychology sessions were offered once a week. The baseline measures conducted over 3 days. Day 1: medical consultation reviewing medical history and measuring vital signs; completed the Physical Activity Questionnaire (IPAQ) (Pinto Guedes et al., 2005) and psychological questionnaires. Day 2, a fasting (~12 h) venous blood sample was collected, anthropometrics including body composition were measured, and food records were completed by participants. Day 3: The adolescents performed the fitness tests.

The a priori sample size calculation, based on initial work (Magnani Branco et al., 2018; Branco et al., 2019), indicated that 9 adolescents per group were sufficient to detect changes in dependent variables with a similar standard deviation (α = 0.05 and β = 80%). The present study was approved by the Local Committee for Ethics in Human Research and was registered in Clinical Trials Registry Platform. This study followed the Declaration of Helsinki. The parents/guardians provided informed consent, and the adolescents signed an assent form agreeing to participate in this research.

### Study Participants

**Figure 1:**
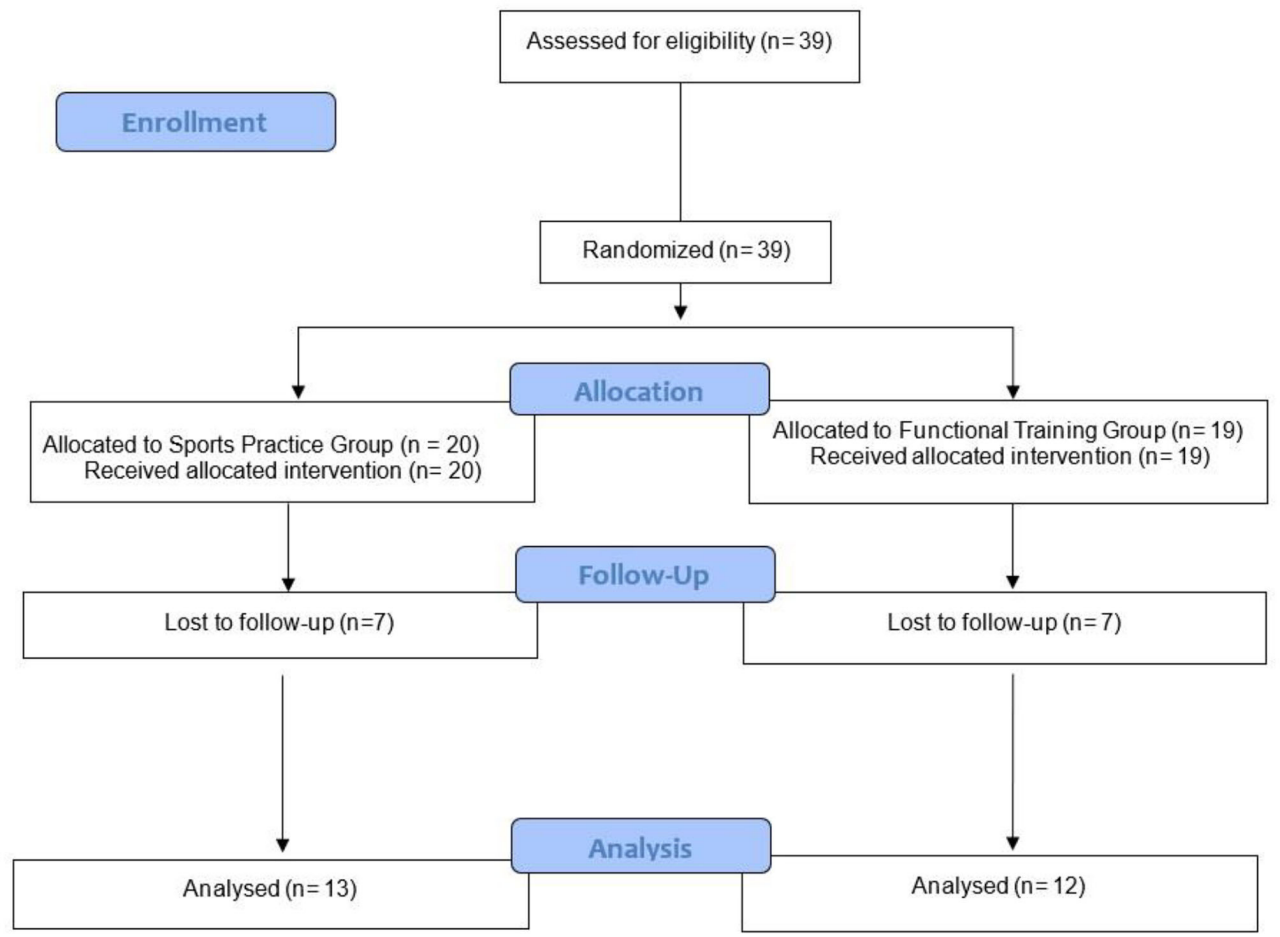
Flowchart of the present study.

Forty-three female adolescents were recruited, but thirty-nine agreed to participate in the present study. The adolescents were randomized into 2 experimental groups: SPG; *n* = 20 and FTG; *n* = 19. However, 7 adolescents dropped out of the SPG, and 7 dropped out of the FTG during the 12-week multi-disciplinary intervention. To be eligible, adolescent girls could not be enrolled in other structured PA programs focusing on weight loss. While encouraged to be physically active, participants were asked to refrain from engaging in structured physical training or dietary programs or interventions outside the university over the course of this study. Further details are presented in CONSORT flow diagram in Figure 1.

### Multi-Disciplinary Intervention Systematization

The 12-week multi-disciplinary intervention included identical theoretical and practical classes occurring 3 times a week; nutrition classes were offered 2 times a week before the PA sessions) and psychology, 1 time a week, before PA. The only difference between intervention groups was the type of exercise, either SPG and FTG, which participants engaged in 3 times a week.

### Physical Activity Intervention

Both physical activity models were equalized by RPE 6-20 proposed by Borg (1982). The activities took place according to Table 1. It is highlighted that the RPE is individual and subjective; that is, the RPE for one adolescent may be different for another during similar exercises. Thus, the adolescents were instructed on the intensity of the RPE before the study, and in all PA sessions. The scale was placed on the wall of the gym so that all adolescents could monitor the intensity of their physical efforts. Table 1 shows the intensity and time per session for both PA groups during a 12-week multi-disciplinary intervention. Table 2 shows training program for functional training group.

**Table 1:** Intensity and time per session during 12 weeks of intervention.

**Intensity**	**Time per session**	**Effort: pause ratio**	**Week**
10–12 a.u.	40 min	30″ by 30″	1st and 2nd
10–12 a.u.	68 min	30″ by 30″	3rd and 4th
12–14 a.u.	40 min	45″ by 15″	5th and 6th
12–14 a.u.	68 min	45″ by 15″	7th and 8th
15–17 a.u.	40 min	30″ by 30″	9th and 10th
15–17 a.u.	68 min	30″ by 30″	11th and 12th

*a.u., arbitrary unity*.

**Table 2:** Training program A and B during 12 weeks of intervention.

	**Exercise**	**Equipment**	**Set**	**Repetition**	**Velocity (C:E)**
**Training program A**					
1	Warming-up–interval jogging	Cones, agility ladder, stopwatch, and whistle	10 min	In accordance with periodization	Only one time
2	Suspended row	TRX	2x	idem	2:2
3	Rope Tsunami	Naval rope	2x	idem	Moderate speed
4	Pull tire	Tire and naval rope	2x	idem	Moderate speed
5	Montain climber	Body weight	2x	idem	Moderate speed
6	Half squat	Body weight	2x	idem	2:2
7	Walk forward and flex your torso by throwing the medicine-ball	Medicine-ball 2 Kg	2x	idem	Moderate speed
8	Up and down the tire alternating right and left	Body weight	2x	idem	Moderate speed
9	Jogging with lateral displacement touching the cones	Cones	2x	idem	Moderate speed
10	Half squat in isometric position abducting thighs with a Swiss ball	Swiss ball	2x	idem	Moderate pressure
11	Crunch	Mattress	2x	idem	2:2
12	Slow jogging	Stopwatch and whistle	5 min	idem	Only one time
13	Stretching	Light stretching	5 min	idem	Only in the end, before ask about RPE
**Training program B**					
1	Warming-up–interval jogging	Cones, agility ladder, stopwatch, and whistle	10 min	In accordance with periodization	Only one time
2	Push up (on knees)	Step	2x	idem	2:2
3	Skip	Body weight	2x	idem	Moderate speed
4	Medicine ball chest throw	Medicine- ball	2x	idem	Moderate speed
5	Front displacement in the agility ladder	Agility ladder	2x	idem	Moderate speed
6	Leg Curl	Bean	2x	idem	2:2
7	Lateral displacement on the agility ladder	Agility ladder	2x	idem	Moderate speed
8	Straight leg deadlift	Medicine-ball or slam-ball	2x	idem	2:2
9	Running with front and rear displacement	Cones	2x	idem	Moderate speed
10	Abduction of legs with lateral displacement using elastic	Elastic	2x	idem	2:2
11	Twisting sit-up	Mattress	2x	idem	2:2
12	Slow jogging	Stopwatch and whistle	5 min	idem	Only one time
13	Stretching	Light stretching	5 min	idem	Only in the end, before ask about RPE

*RPE, rating of perceived exertion; TRX, suspension training*.

PA performed by SPG was divided into three sports, one sport per month: basketball, handball, and futsal. The different sports were conducted in the following manner: warm-up *via* interval jogging with the same equipment used in the functional training group for 10 min; carrying out specific activities, such as passing, dribbling, small-sided games and recreational games with mediated intensity by the physical trainer; and slow jogging and stretching using the same time and intensity presented in Table 1. The RPE was used to control intensity and time per session to control volume, in accordance with Branco et al. (2018, 2019).

### Nutrition Intervention

The main objective of the nutritional intervention was to emphasize the following: (a) the importance of macronutrients and micronutrients and their impact on adolescent health; (b) the food pyramid; (c) the importance of fiber consumption and the soluble and insoluble fibers; (d) the energy density of foods; (e) the difference between light and diet foods; (f) eating disorders; (g) differences between minimally processed, processed and ultra-processed foods; (h) amounts of salt, sugar, and fat in foods; (i) consuming foods for health and quality of life using food re-education as an instrument; and (j) methods for preparing healthy foods. Meetings were held twice a week, with an average duration of 1 h (Magnani Branco et al., 2018; Branco et al., 2019).

### Psychological Intervention

The psychological intervention was developed to help adolescents improve the behavioral change process, the relationship with food consumption, skills, motivation, and self-confidence (MacKinnon et al., 2010). Cognitive and behavioral strategies were used to improve the quality of food consumption among the adolescents, which included anxiety control, self-monitoring, identifying negative feelings, and health education. Furthermore, strategies were implemented to improve the assessment of body self-image. The psychological intervention was divided into five subject matter areas: eating habits, self-monitoring, control of anxiety, negative feelings, and health education. The cognitive-behavioral intervention component was delivered in a group setting held weekly for 1 h as performed in previous studies with similar designs (Magnani Branco et al., 2018; Branco et al., 2019).

### Body Composition and Anthropometry

Bioelectrical impedance (BIA) was used to measure body composition using an eight-tailed four-quadrupole multifrequencial body composition apparatus (In Body model 570® Body Composition Analyzers; Seoul, South Korea). On the first day of evaluation, all participants received an informative document containing the protocol used to perform the assessment, which included (a) 12-h fasting, (b) no moderate or vigorous physical exercise within 24-h preceding the test, (c) urination and evacuation for the evaluation, (d) the absence of metallic objects during the evaluation, (e) postponement of the assessment in menstruating participants until after their period, (f) at least 8 h of sleep, and (g) no ingestion of caffeinated beverages or foods during the 12-h prior to the measurement (Heyward, 2001; Branco et al., 2018). The following BIA variables evaluated: body weight, body mass index (BMI), musculoskeletal (MME) mass, fat mass (FM), and body fat percentage (BF).

Weight and height were measured using an electronic scale and a stadiometer (Filizola® São Paulo, Brazil). Their capacities are 200 kg (accuracy = 100 g) and of 2 m (accuracy = 0.1 cm), respectively.

### Physical Fitness Assessment

Before the measurements were obtained, all adolescents were familiarized with the sum of maximal isometric handgrip strength (SMIHS), maximal isometric lumbar-traction strength (MILTS), abdominal strength/endurance, flexibility, and aerobic fitness tests.

#### MIHS

A Jamar dynamometer (Asimow Engineering; Los Angeles, CA, United States) was used to evaluate MIHS. The subject remained standing while holding the device close to her body with her arm extended and a neutral grip. Following a signal by the evaluator, the adolescent squeezed the dynamometer as hard as possible while maintaining isometric contractions for 3 to 5 s. The measurements were performed with both hands. The sum of the right and left hand (sum of maximum isometric handgrip strength of right and left hand–SMIHS in the statistical analysis) after three attempts on each side was used to determine the maximum isometric strength of the handgrip. A 60 s interval separated each attempt (Branco et al., 2017).

#### MILTS

A Kratos dynamometer (Kratos Industrial Equipment, Model DS® São Paulo, Brazil) was used to evaluate MILTS. The teenager walked with both feet on the device with her, trunk extended and flexed ~120°, head and neck aligned with the trunk, and fingers (holding the bar) positioned in front of the patella bone. The maximum contraction over 3 to 5 s was recorded, and the highest value of the measurement after three attempts with 60 s of rest between attempts was recorded (Branco et al., 2017).

#### Abdominal Strength/Resistance

Abdominal strength was assessed by testing the maximum number of repetitions in 60 s of trunk flexion (i.e., sit-ups). This test followed the methodology proposed by the Fitness Gram Reference Guide (Plowman and Meredith, 2013).

#### Flexibility Test

Flexibility assessment was performed using the sit-and-reach test, during which participants were asked to sit with legs outstretched and then reach forward to try to achieve the greatest distance using both hands; one above the other. This test followed the methodology proposed by the Fitness Gram Reference Guide (Plowman and Meredith, 2013).

#### Aerobic Fitness

The multistage fitness test, proposed by Léger et al. (1988), was used to measure aerobic capacity and provide an estimate for maximal oxygen consumption (VO_2_max). This field test has 21 stages; the initial running velocity is 8.5 km/h, which increases by 0.5 km/h per stage. The VO_2_max was calculated using the following equation:

$$\begin{array}{l} VO_{2}\max=31.025+\left(3.288\;*\;X\right)-\left(3.248\;*\;A\right)+\left(0.1536\;*\;A\;*\;X\right)\; \end{array}$$

Where X = velocity in the stage reached, and A = age in years.

### Training Monitoring

To monitor the physical training sessions, several non-invasive, and practical instruments were used by the physical trainer. Prior to each training session, the rating of the perceived recovery (RPR) scale as proposed by Laurent et al. (2011), the RPE-session as proposed by Foster et al. (2001), and the internal training load (ITL) were assessed. The RPR indicates the recovery status and is used before each training session. The scale is comprised of numerical values ranging from 0 a.u. (very poor recovery/extremely tired) to 10 a.u. (very good recovery/extremely well-disposed). The RPE-session was performed 30 min after the end of the training sessions following the original recommendations of Foster et al. (2001). This scale is comprised of values ranging from 0 a.u. (extremely light effort) to 10 a.u. (extremely heavy stress). The ITL was calculated by multiplying the value of the RPE by the duration of the training session in minutes. The adolescents were familiarized with the scales before and during the interventions. The scales were administered to the participants, and explanations were provided during the theoretical classes and before the PE sessions.

### Instruments Used to Assess the Psychological Variables

#### Body Image Assessment

To assess dissatisfaction with body image, the Body Shape Questionnaire (BSQ) was used. This tool was initially validated by Cooper et al. (1987) and validated for the Brazilian population by Di Pietro and Da Silveira (2008). The BSQ consists of 34 questions about dissatisfaction with body image and concern over body measurements. Each question is assigned a value on a scale of 1 to 6, with 1 being never, and 6 being always. The higher scores are reflective of higher body dissatisfaction. Based on the score, individuals are classified as satisfied (81 and 110 points) and dissatisfied (above 111 points) with body image.

#### Assessment of Eating Attitudes

To assess eating attitudes, we used the Eating Attitudes Test (EAT-26), containing twenty-six self-report questions. In Brazil, the translation of the EAT-26 was performed by Nunes et al. (1994), and validated in a population of adolescents by Fortes et al. (2016). The evaluation of EAT-26 responses are made by assigning three scores to each item, where the most extreme anorexic response was marked (“always”), two scores for the second most extreme answer (“very often”) and a score for the third most extreme (“frequently”); less extreme answers are not scored. Question number 4 presents a particularity, as the scoring is done in reverse; that is, “sometimes” is equivalent to 1 point, “rarely” is equivalent to 2 points, and “never” is equivalent to 3 points. However, only question 4, that uses inverse scoring, allows for no score. If the total score found was >21, EAT-26 was considered positive (EAT-26+) and confirmed the presence of pathological eating attitudes and risk of developing eating disorders.

#### Self-Esteem Assessment

To assess the subjects' self-esteem the Self-Esteem Scale, developed by Rosenberg (1989), was used. This scale is a one-dimensional measure that consists of ten statements related to a set of feelings of self-esteem and self-acceptance that assesses global self-esteem. The items are answered on a Likert scale of four points ranging from strongly agree, agree, disagree, and strongly disagree. This study used the adapted Portuguese version (Hutz and Zanon, 2011).

#### Anxiety Assessment

The Hamilton Scale was used for the anxiety assessment (HAM–A) (Hamilton, 1959). This instrument examines 14 groups of symptoms, subdivided into two groups, seven related to mood anxiety symptoms, and seven related to physical anxiety symptoms. To analyze the results of this study, we differentiated psychic anxiety (the group of mood anxious symptoms) and somatic anxiety (the group of physical symptoms). Each item is evaluated according to a scale ranging from 0 to 4 in intensity (0 = absent; 2 = mild; 3 = medium; 4 = maximum). The sum of the scores obtained for each item resulted in a total score ranging from 0 to 56. If the number of symptoms is relatively high, symptom counting becomes an instrument– useful, reliable, and good validation quantifier (Pasquali, 1998).

#### Depression Assessment

The PHQ-9 instrument was originally developed by Kroenke, Spitzer, and Williams (Kroenke et al., 2001). The instrument has been validated for the Brazilian population by Santos et al. (2013) and adapted for adolescents. The PHQ-9 is characterized as being an instrument of relatively fast application, containing 9 questions that assess the presence of each of the symptoms for episodes of major depression, as described in the Diagnostic and Statistical Manual of Mental Disorders (DSM-IV). The nine symptoms consist of depressed mood, anhedonia (loss of interest or pleasure in doing things), problems with sleep, tiredness or lack of energy, change in appetite or weight, feelings of guilt or worthlessness, concentration problems, feeling slow or restless, and thoughts of suicide. The frequency of each symptom in the past 2 weeks is evaluated on a Likert scale from 0 to 3 corresponding with “no time” answers, “several days,” “more than half the days,” and “almost every day,” respectively. The questionnaire also includes a tenth question that assesses the interference of these symptoms in the performance of daily activities, such as work and study. As a measure of severity, the PHQ-9 score can vary from 0 to 27, as each of the 9 items can be scored from 0 (nothing) to 3 (almost every day).

### Dietary Record Assessment

The adolescents were instructed to keep a 3-day food diary. In the food diary they were asked to indicate all foods and beverages consumed throughout the day, record the size of portions consumed and include recipes, ingredients, and methods of preparation when relevant (Magnani Branco et al., 2018; Branco et al., 2019). They were also instructed to include details, such as the addition of salt, sugar, oil, and sauces, and whether the food or drink consumed was regular, diet, or light. For the best estimation of the portion size, they used home measurements (Slater et al., 2003). It is important to note that there was no dietary planning or feeding control, so it was suggested that adolescents change their dietary habits according to theoretical and practical classes on nutrition and psychological interventions. Subsequently, responses resulting from the dietary records were analyzed using the software Avanutri 2004 (Avanutri Equipamentos de Avaliação Ltd.; Três Rios, Brazil). Using the daily dietary record, the daily kilocalories intake of the adolescents was calculated, and the values pre- and post-intervention were compared. The comparative analysis used the mean values of the 3 days of dietary records. Individual values of 2 interspersed days of records during a weekday and 1 weekend day of caloric intake were used (Kcal.d^−1^) (Magnani Branco et al., 2018; Branco et al., 2019).

### Biochemical Tests

Blood samples were collected by means of venous blood in the morning after 12 h of fasting. Vacuum tubes with a clot activator tube–silica (Becton Dickinson Vacutainer®, Plymouth, United Kingdom) were used for blood collections. Blood samples were centrifuged at 3,600 rpm for 11 min at a controlled temperature (24°C), and serum was frozen at −80°C allowing for post-study analysis. Fasting levels of serum glucose, total cholesterol (TC), high-density lipoprotein (HDL-c), low-density lipoprotein (LDL-c), and triglyceride (TG) were analyzed by an independent biomedical team blinded to group assessment. All analyses were performed in triplicate. For fasting glucose, TC, and LDL-c, the enzymatic-colorimetric method (Trinder) was used. For TG, the enzymatic-colorimetric method (Trinder) with a lipid-bleaching factor was used, while for HDL-c, the selective surfactant method was used. For the biochemical analyses, a Siemens equipment (Advia 1800 Chemistry Analyzer®, Siemens Healthcare Diagnostics; Illinois, United States) was used.

### Statistical Analyses

The normality of the data was tested using the Shapiro-Wilk test, and homogeneity was tested using Levene's test. After confirming these parameters, the data were expressed as the mean ± standard deviation. A 2-way analysis of variance (ANOVA) was performed, and when a difference was detected, Bonferroni's *post hoc* test was applied. Mauchly's test of sphericity was used to test for equality between levels of independent variables, as was the Greenhouse-Geisser correction, if necessary. A *p* < 0.05 was considered significant for all analyses. In addition, to the ANOVA results, the effect size was calculated using Cohen's d (Cohen, 1988) and interpreted as follows: 0.20 (small effect), 0.50 (moderate effect), and 0.80 (large effect). Furthermore, the partial eta-squared (η^2^) was calculated with the following classification: 0.01 (small effect), 0.06 (moderate effect), and 0.14 (large effect) according to Cohen (1988). All statistical analyses were performed using the statistical package SPSS 22.0 (IBM, Inc.; United States).

## Results

The age of participants did not differ between groups. SPG presented 12.8 ± 3.0 years old, whereas the FTG presented 12.9 ± 3.2 years old.

**Figure 2:**
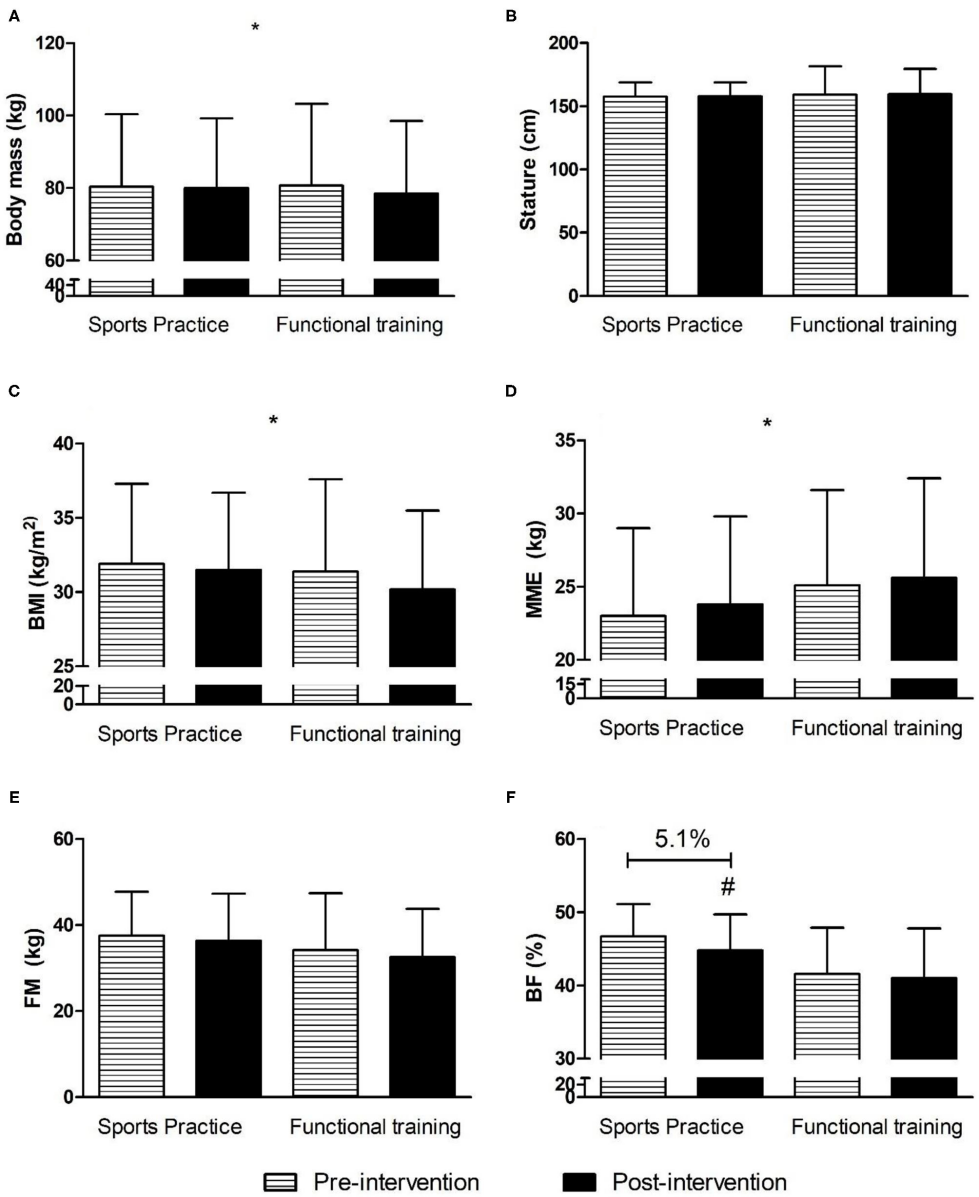
Anthropometric and body composition variables before and after 12 weeks of interdisciplinary intervention. Data is expressed by mean and standard deviation; *time effect with *p* < 0.05; ^#^group × time interaction for body fat in Sports Practice Group with *p* < 0.05; BMI, body mass index; MME, musculoskeletal mass; FM, fat mass; BF, body fat. **(A)** Body mass in pre- and post-intervention for Sports Practice Group and Functional Training Group; **(B)** Stature in pre- and post-intervention for Sports Practice Group and Functional Training Group; **(C)** Body mass index in pre- and post-intervention for Sports Practice Group and Functional Training Group; **(D)** musculoskeletal mass in pre- and post-intervention for Sports Practice Group and Functional Training Group; **(E)** Fat mass in pre- and post-intervention for Sports Practice Group and Functional Training Group; **(F)** Body fat percentage in pre- and post-intervention for Sports Practice Group and Functional Training Group.

Figure 2 shows the anthropometric and body composition variables of the adolescents participating in the present study.

For body mass, there was only a time effect [*F*_(1, 26)_ = 5.3; *p* = 0.029; η^2^ = 0.17; large] with lower values observed after the intervention (*p* = 0.029). For BMI, there was only a time effect [*F*_(1, 26)_ = 11.2; *p* = 0.002; η^2^ = 0.30; large] with lower values observed after the intervention (*p* = 0.002). For MME, there was only a time effect [*F*_(1, 26)_ = 11.5; *p* = 0.002; η^2^ = 0.30; large] with higher values observed after the intervention (*p* = 0.029). For BF, there was a group by time interaction effect [*F*_(1, 26)_ = 6.9; *p* = 0.014; η^2^ = 0.20; large] with a *post-hoc* test identifying lower values for SPG in the post-intervention period (*p* = 0.04; *d* = 0.38; small). For height, there was no difference between group, time, or interaction (*p* > 0.05). Table 3 shows the physical fitness related to the health of the adolescents participating in the present study.

**Table 3:** Physical fitness related to the health during 12 weeks for the two experimental groups.

**Variables**	**Sports Practice Group**			**Functional Training Group**		
	**Pre**	**Post**	***Cohen's d***	**Pre**	**Post**	***Cohen's d***
MIHS (kg)	46.0 ± 14.8	45.9 ± 14.7	0.00	49.3 ± 18.3	49.0 ± 17.8	−0.01
MILTS (kg)	51.5 ± 21.3	51.9 ± 21.1	0.01	63.5 ± 33.4	73.7 ± 37.6	0.30
Abdominal strength/endurance (reps)	21 ± 4	21 ± 5	0.00	25 ± 5	25 ± 9	0.00
Flexibility (cm)	30 ± 10	32 ± 7	0.20	32 ± 7	31 ± 8	−0.14
VO_2_max (mL/kg/min)^*^	35.3 ± 10.5	38.6 ± 5.6	0.31	37.1 ± 5.2	38.5 ± 4.0	0.26

Data is expressed by mean and ± standard deviation;

* *time effect with p < 0.05. MIHS, maximal isometric handgrip strength (sum of right and left hands); MILTS, maximal isometric lumbar-traction strength*.

**Figure 3:**
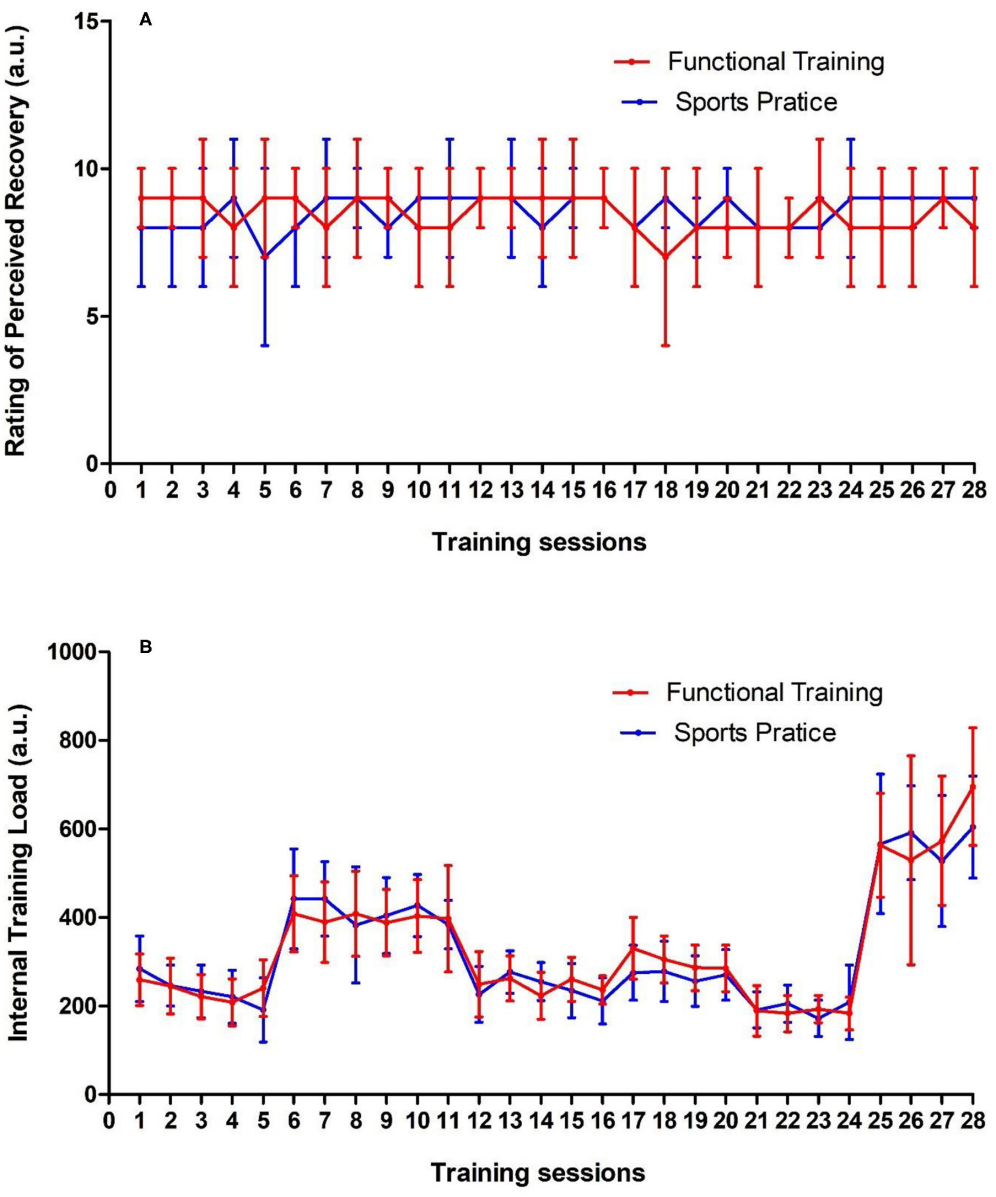
**(A)** Rating of perceived recovery and **(B)** Internal training load of groups throughout of 12 weeks of interdisciplinary intervention. Data is expressed by mean and ± standard deviation; a.u., arbitrary unity.

For SMIHS, MILTS, abdominal strength/endurance, and flexibility, there were no group, time, or interaction effects (*p* > 0.05). For VO_2_max, there was a time effect [*F*_(1, 27)_ = 7.7; *p* = 0.009; η^2^ = 0.22; large] with higher values post-intervention (*p* = 0.009). Figure 3 shows the RPR and ITL of both groups during the 12-week intervention.

Table 4 presents the health mental responses during the 12-week intervention of the adolescents participating in the present study.

**Table 4:** Health mental responses of the adolescents during 12 weeks of intervention.

**Variables**	**Sports practice group**			**Functional training group**		
	**Pre**	**Post**	***Cohen's d***	**Pre**	**Post**	***Cohen's d***
Body image	108 ± 41	96 ± 41	−0.29	83 ± 30	77 ± 35	−0.20
Eating attitudes	11 ± 8	12 ± 8	0.12	11 ± 8	15 ± 14	−0.50
Self esteem	3 ± 0	3 ± 0	0.00	2 ± 0	3 ± 0	0.00
General anxiety	1 ± 1	1 ± 1	0.00	1 ± 0	1 ± 1	0.00
Psychic anxiety	1 ± 1	1 ± 1	0.00	1 ± 1	1 ± 1	0.00
Somatic anxiety	1 ± 1	1 ± 1	0.00	0 ± 0	1 ± 1	0.00
Depression	10 ± 6	10 ± 5	0.00	7 ± 6	7 ± 6	0.00

*Data is expressed by mean and ± standard deviation*.

**Figure 4:**
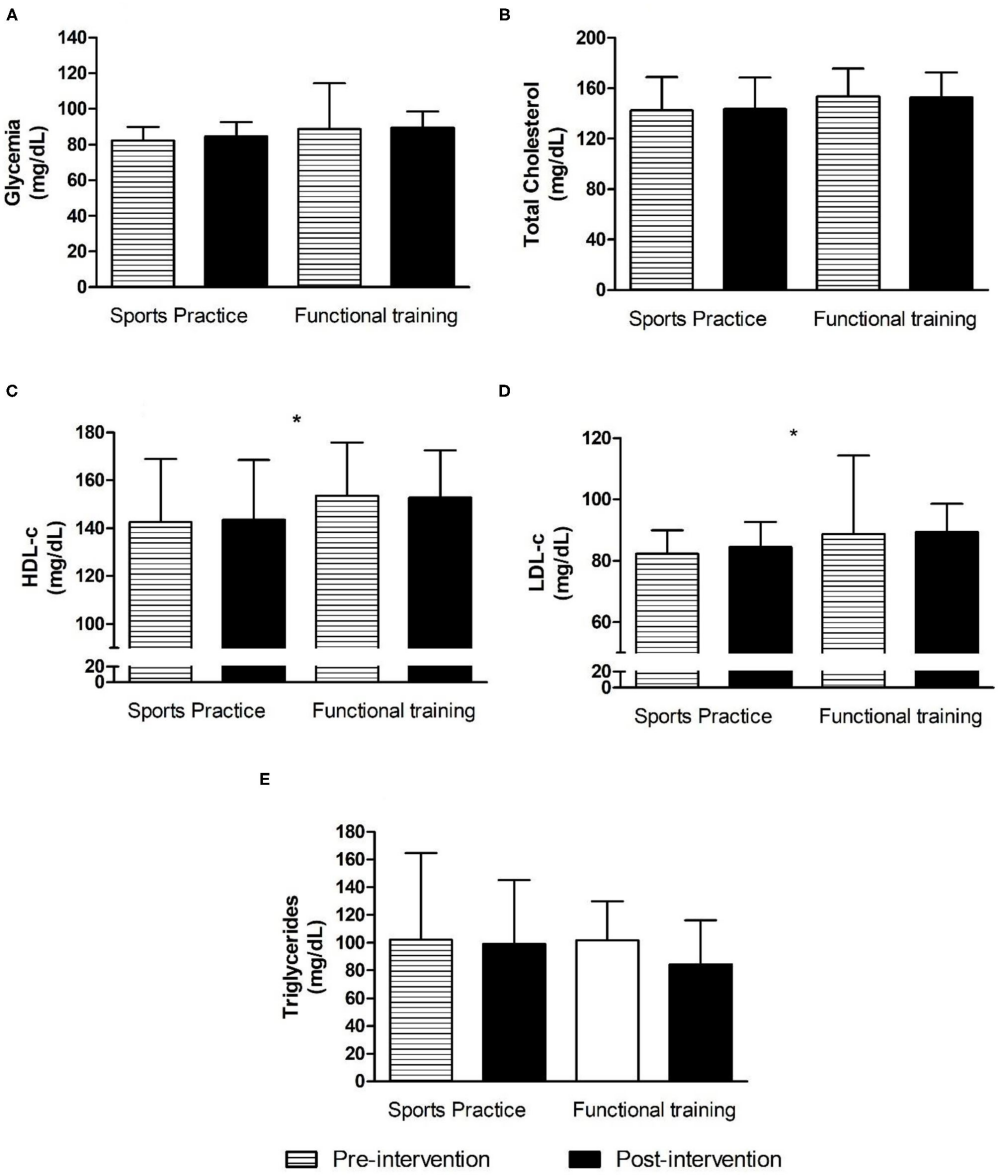
Biochemical variables before and after 12 weeks of interdisciplinary intervention. Data is expressed by mean and ± standard deviation; *time effect with *p* < 0.05. HDL-c, high-density lipoprotein; LDL-c, low-density lipoprotein. **(A)** Glycemia in pre- and post-intervention for Sports Practice Group and Functional Training Group; **(B)** Total Cholesterol in pre- and post-intervention for Sports Practice Group and Functional Training Group; **(C)** High-density lipoprotein in pre- and post-intervention for Sports Practice Group and Functional Training Group; **(D)** Low-density lipoprotein in pre- and post-intervention for Sports Practice Group and Functional Training Group; **(E)** Triglycerides in pre- and post-interventions for Sports Practice Group and Functional Training Group.

There were no significant group, time, or interactions for IPAQ over the 12-week intervention period (*p* > 0.05). The only difference observed was an increase in the level of PA on Mondays, Wednesdays, and Fridays, which was expected based on the intervention design. For body image, eating attitudes, self-esteem, general anxiety, psychic anxiety, somatic anxiety, and depression there were no group, time, or interaction effects (*p* > 0.05). Figure 4 shows the fasting glucose and lipid profile of the adolescents participating in the present study.

For fasting glucose, TC, and TG, there were no group, time, or interaction effects (*p* > 0.05). For HDL-c, there was only a time effect [*F*_(1, 23)_ = 16.23; *p* = 0.0005; η^2^ = 0.41; large], with higher values noted post-intervention (*p* = 0.004). Finally, for LDL-c, there was also a time effect [*F*_(1, 23)_ = 23.93; *p* = 0.0000; η^2^ = 0.51; large], with lower values observed after the post-intervention (*p* < 0.001). Food diary assessments showed no difference between groups, and no time or interaction effects during the 12-week intervention. The same was observed for RPR and ITL during the same measurement period (*p* > 0.05).

## Discussion

The main objective of the present study was to investigate the effects of two different modes of PA, matched by RPE, on anthropometric, body composition, physical, cardiometabolic risk, and psychological responses.

As such, the following responses were observed for anthropometrical variables: (1) a post-intervention reduction of body mass and BMI in both experimental groups; (2) increased musculoskeletal mass in both experimental groups; (3) a group by time interaction effect with lower values for SPG;

The responses for fitness assessments: (1) no significant differences observed for SMIHS, MILTS, abdominal strength/endurance, and flexibility; (2) increased VO_2_max in both experimental groups; (3) no significant differences for RPR and ITL in the two experimental groups;

The responses for psychological health parameters: (1) a small effect for body image assessment with lower values; (2) for the other psychological questionnaires there were no differences;

The responses for cardiometabolic risk parameters: (1) a significant increase in HDL-c in both experimental groups; (2) a significant reduction in LDL-c in both experimental groups.

However, no significant differences were observed for the other variables analyzed in the present study. Therefore, based on the results presented, the study's hypothesis that the responses in anthropometrical, physical fitness, psychological and cardiometabolic risk are similar, as the two intervention models are equalized by RPE, was confirmed.

Although reductions in body mass and BMI have been observed after the intervention period, it is noteworthy that neither anthropometric measures are considered the gold standard for measuring possible differences after the multi-professional intervention, with a focus on the treatment of obesity in adolescents (Magnani Branco et al., 2018; Branco et al., 2019). Such responses may be related to increase MME and reduce FM during interventions in adolescents (Branco et al., 2019). Therefore, BMI is not a sensitive indicator for detecting possible differences in the body composition of adolescents (Tylka et al., 2014), since the instrument is limited to being an anthropometric index (Magnani Branco et al., 2018; Branco et al., 2019). Although the limitations of BMI were previously highlighted, Melnyk et al. (2009) pointed out that a reduction in BMI, reductions in symptoms of anxiety and depression, a reduction in triglycerides, and an increase in HDL-c are all significant contributors for improving the quality of life of adolescents who are overweight or obese. However, the inefficiency of an approach centered in the body weight should be noted, given that the focus of this method tends to be stigmatized in health care (Telama, 2009). We recognize that the reduction in body weight and BMI should be interpreted with caution. Although reductions in fat mass, waist circumference, and BMI z-score are important clinically, we must also focus programs on increasing cardiorespiratory fitness, lean mass, and improving the total domain of Health-Related Quality of Life Assessment in adolescents who are overweight or obese (Nardo Junior et al., 2018).

The increase in MME is a fundamentally relevant outcome point of the present study. Thus, according to the preference of adolescents, intervention models in PA that result in greater adherence and, consequently, better results in the medium and long term, regarding body composition and VO_2_max can be designed. The increase in MME tends to increase people's total energy expenditure by 13 kcal/kg/day (McClave and Snider, 2001), contributing to the weight loss process. We know encouragement and habitual participation in PA in childhood and adolescence is associated with greater adherence to PA in adulthood, and a factor called physical activity tracking (Telama, 2009). Also, worth noting is that physical inactivity accounts for about 6% of deaths in the world and is associated with increased blood pressure, hyperglycemia, and obesity—all of which have high public health costs (Wei et al., 1999). Thus, the benefits of encouraging PA become substantial in adolescent age to promote healthy longevity and reduce public health spending.

Our intervention models equalized by volume, intensity, and muscle groups exhibited a tendency to present similar responses in terms of anthropometric parameters, body composition, physical fitness related to health as well as lipid, glycemic, and liver enzyme profiles (Magnani Branco et al., 2018; Branco et al., 2019). However, only the SPG intervention results in a reduction in body fat percentage. The divergence between the responses of the body fat percentage can be justified using cyclic exercises vs. acyclic exercises over the 12-week intervention, despite the standardization of the intensity of the sessions during the intervention period. One point that was not possible to equalize was the effort-pause ratio of the different models of PA due to differing protocols. Consequently, it is believed that the SPG may have presented a higher energy expenditure than the FTG due to the specificity of the activities developed in each group. As mentioned, the FTG group presented an effort-pause ratio between 30″ by 30″ and 45″ by 15″ and intensity between 10 a.u. and 17 a.u. The SPG did not present a defined effort-pause ratio. Only the volume and intensity of the sessions were controlled, which was similarly done in the FTG. Therefore, based on the absence of differences for the 3-day non-consecutive food records and the IPAQ, it is believed that the SPG sessions resulted in greater energy expenditure when compared to the FTG. Previous evidence indicates that the use of different types of equipment to perform physical exercises even with the same RPE may present greater or lesser energy expenditure, depending on what is performed (Zeni et al., 1996; Moyna et al., 2001). From this perspective, Moyna et al. (2001) pointed out that physical exercises involving greater body mass tend to expend greater amounts of energy, even when performed by the same RPE. This question may explain the group by time interaction effect with lower values for BF in SPG. However, new studies with control of energy expenditure during and after the different models of PA could elucidate the responses observed in the present study.

The absence of differences in testing such as SMIHS, MILTS, abdominal strength/endurance and flexibility may be explained by the choice of the physical fitness tests. Test selection could be considered a limitation in that the physical fitness tests may not have been specific enough to assess possible changes after the intervention period, as has already been discussed in sports training (Andreato and Branco, 2016). Thus, we propose using the full set of testing pieces as suggested by the Fitnessgram/ActivityGram Reference Guide (Plowman and Meredith, 2013) and not partial, as supported by previous longitudinal studies focusing on the multi-disciplinary treatment of obesity in children and adolescents. In turn, an increase in VO_2_max was observed in both models of PA. The literature indicates that obese adolescents have less cardiorespiratory fitness than non-obese adolescents (Wei et al., 1999). Insufficient cardiorespiratory fitness increases the risk of cardiovascular disease (Magnani Branco et al., 2018; Branco et al., 2019). Besides, Nardo Junior et al. (2018) point out that the improvement of cardiovascular health can be considered a key component of adolescent health. In view of this, and considering that the two intervention models raised VO_2_max, the choice of the type of PA will also need to consider the adolescents' wants and needs in order to maintain adherence to the health-promoting process and increase the quality of life.

Regarding mental health, Alberga et al. (2019) pointed out that adolescents with obesity are less likely to adhere to physical exercise, and body image and mood need to be tracked to adjust the physical exercise schedule. Therefore, physical exercises with the highest adherence will be those that the adolescents exhibit the greatest pleasure and satisfaction doing during the practice. In turn, previous evidence suggests that 12 weeks of multi-professional and interdisciplinary interventions are insufficient to promote changes in the behavioral parameters of overweight or obese adolescents (Costa et al., 2019). According to the authors, interventions focused on improving aspects related to adolescents' mental health could involve group activities with parents or guardians and activities with adolescents only. Therefore, the interrelationship between adolescents, family, and health professionals tend to present more promising results when compared to the approach performed in isolation with adolescents.

On the other hand, Freitas et al. (2017) who, in a similar experimental design using only aerobic exercises 3 × a week, nutritional counseling 1 × a week, and psychological counseling 1 × a week, obtained a significant increase in parameters related to the quality of life. In the Freitas study, a generic instrument was used to assess the quality of life using the SF-36 questionnaire. Improvements were identified in the following areas: functioning and physical appearance increased general perception of health and vitality, improvement of emotional and mental health, and consequent increase in the average of the dimensions of the applied questionnaire. However, as a central limitation, one can list the non-specificity of the questionnaire used to measure specific aspects of the adolescents' mental health such as body image, eating attitudes, general, psychological, and somatic anxiety, as well as depression. The only finding observed was a slight reduction in dissatisfaction with body image in both groups after the intervention period. It is assumed that the reduction of dissatisfaction with the body image of the adolescents is related to the homogeneity of the intervention groups; that is, the treatment groups were composed of adolescents of the same sex, overweight or obese, with the same level social and the same age group. With such a characteristic, it is assumed that the adolescents accepted their own body image more positively. In this same line of reasoning, Costa et al. (2019) identified that adolescents participating in a 12-week multi-professional intervention focusing on the treatment of obesity showed significant reductions for the BSQ questionnaire, precisely the question: “being with thin people of the same sex as you, makes you feel worried about the relation to your physique?” As a result, it is believed that the establishment of homogeneous groups may contribute to the self-acceptance of adolescents' body image.

Positive changes were observed in two variables of the lipid profile of the two experimental groups, namely an increase in HDL-c and a reduction in LDL-c. Mann et al. (2014), in a robust review of the literature, pointed out that the combination of resistance exercises of moderate intensity in the form of a circuit can be beneficial for increasing HDL-c and reducing LDL-c. Similar designs have already shown divergent results regarding the increase in HDL-c in adolescents during the same intervention period (Magnani Branco et al., 2018; Branco et al., 2019). In this aspect, Branco et al. (2018, 2019) pointed out that the elevation of HDL-c is associated with the incorporation of the ideal volume and intensity of predominantly aerobic stimuli. Similarly, in the present study, both models of physical activity showed several predominantly aerobic actions, a factor which justifies the increase in HDL-c after the 12- week intervention. In opposition, no significant differences were identified for fasting glucose, total cholesterol, and triglycerides. On average, the glycemic values before and after the intervention period were within the standard cutoff points (Suglia et al., 2018). Thus, the absence of differences in fasting glucose was not a surprise since previous studies with a multi-disciplinary and interdisciplinary approach focusing on the treatment of obesity in adolescents identified similar results (Magnani Branco et al., 2018; Branco et al., 2019). The measurement of total cholesterol without the analysis of fractions becomes insufficient to evaluate the responses of a multi-disciplinary intervention. Such points are justified by possible increases in HDL-c, concomitantly, with reductions in LDL-c. The maintenance of triglyceride values can be explained by the absence of differences in eating habits inferred by the responses before and after filling in the food record for three non-consecutive days in addition to the cutoff points being within normal cutoff values (Nordestgaard et al., 2013).

Despite the limitations observed, the responses observed in the study suggest that both models of PA provide similar health benefits, and the choice of the proposed protocol may include the resources available for the practice of PA as well as adolescents' choices. Therefore, it would not be possible to control the intensity of the sessions of the two groups *via* HR. Thus, the RPE is the most acceptable method, although limitations are observed. Finally, the use of RPE to control physical exercise sessions may be an interesting strategy to control training intensity (Haddad et al., 2017). In this sense, Haddad et al. (2017) in a robust systematic review confirmed the reliability and internal consistency of the use of perceptual scales to control the intensity of physical and sports activities in different age groups, such as children and adolescents with different levels of experience.

## Conclusions

Based on the results, it appears that both models of PA promoted significant reductions in body mass, BMI, dissatisfaction with body image (*via* effect size), and LDL-c as well as a significant increase in MME, VO_2_max and HDL-c and an interaction with reduced body fat percentage in the SPG group in the post-intervention period. No significant differences were observed in the other variables analyzed. Thus, the choice for the PA protocol should consider the enjoyment factor and subsequent adherence of adolescents in the process of multi-disciplinary treatment of obesity.

## Data Availability Statement

The raw data supporting the conclusions of this article will be made available by the authors, without undue reservation.

## Ethics Statement

The studies involving human participants were reviewed and approved by University Center of Maringa under number: 2,505.200/2018 and Clinical Trial Registry under number: RBR 45ywtg (https://ensaiosclinicos.gov.br/rg/RBR-45ywtg). Written informed consent to participate in this study was provided by the participants' legal guardian/next of kin.

## Author Contributions

BB, LO, SB, CA, and KA conceived and planned the experimental design. BB and KA conducted the experiments and wrote the manuscript. BB, IM, and KA analyzed the data and revised final version of the manuscript. All authors contributed to the article and approved the submitted version.

## Conflict of Interest

The authors declare that the research was conducted in the absence of any commercial or financial relationships that could be construed as a potential conflict of interest.
